# Efficacy of an Oral Rehydration Solution Enriched with *Lactobacillus reuteri DSM 17938* and Zinc in the Management of Acute Diarrhoea in Infants: A Randomized, Double-Blind, Placebo-Controlled Trial

**DOI:** 10.3390/nu10091189

**Published:** 2018-09-01

**Authors:** Maria Maragkoudaki, George Chouliaras, Antonia Moutafi, Athanasios Thomas, Archodoula Orfanakou, Alexandra Papadopoulou

**Affiliations:** Division of Gastroenterology and Hepatology, First Department of Pediatrics, University of Athens Children’s Hospital “Agia Sofia”, Thivon and Papadiamantopoulou, 11527 Athens, Greece; mariamariaki@gmail.com (M.M.); georgehouliaras@msn.com (G.C.); tania.moutafi@yahoo.com (A.M.); nassos.thomas@gmail.com (A.T.); adaorfanakou@yahoo.gr (A.O.)

**Keywords:** acute gastroenteritis, children, *Lactobacillus reuteri*, oral rehydration solution, probiotics, zinc

## Abstract

The efficacy of oral rehydration solution (ORS) enriched with *Lactobacillus reuteri DSM 17938* and zinc in infants with acute gastroenteritis, is poorly defined. The aim of this double-blind, randomized, placebo-controlled study, was to assess the efficacy of an ORS enriched with *Lactobacillus reuteri DSM 17938* and zinc (ORS^+^Lr&Z) in well-nourished, non-hospitalized infants with acute diarrhoea. Fifty one infants with acute diarrhoea were randomly assigned to receive either ORS^+^Lr&Z (28 infants, mean ± SD age 1.7 ± 0.7 years, 21 males), or standard ORS (ORS^−^Lr&Z; 23 infants, mean ± SD age 1.8 ± 0.7 years, 16 males). Stools volume and consistency were recorded pre- and posttreatment using the Amsterdam Infant Stool Scale and were compared between the two groups, as well as lost work/day care days, drug administration and need for hospitalization. Both groups showed reduction in the severity of diarrhoea on day two (*p* < 0.001) while, all outcomes showed a trend to be better in the ORS^+^Lr&Z group, without reaching statistical significance, probably due to the relatively small number of patients. No adverse effects were recorded. In conclusion, both ORS were effective in managing acute diarrhoea in well-nourished, non-hospitalized infants. ORS enriched with *L. reuteri DSM 17938* and zinc was well tolerated with no adverse effects.

## 1. Introduction

Oral rehydration solution (ORS) is recommended in infants and children with acute diarrhoea for the treatment or the prevention of dehydration [1]. Zinc supplementation is beneficial in infants and children with acute diarrhoea living in developing countries [2], however, its efficacy in those living in developed countries is poorly defined. Furthermore, selected strains of probiotics have been shown to reduce the duration and the severity of diarrhoea in children with acute diarrhoea and the effect is greater if the probiotics are given within 60 hours from the onset of symptoms [3,4,5,6,7,8]. *Lactobacillus reuteri* (*L. reuteri*) *ATCC 55730* was reported to have a beneficial effect in reducing the duration and severity of acute gastroenteritis of both bacterial and viral (rotavirus) origin in infants and toddlers aged 6–36 months [6,7,8]. However, the above strain was found to carry transferable resistance traits for tetracycline and lincomycin, and for this reason it was replaced by a new strain—*L. reuteri DSM 17938*—by removal of two potentially transferable plasmid-borne resistances. Furthermore, *L. reuteri DSM 17938* was assessed in hospitalized and non-hospitalized children with acute diarrhoea with varied results. The administration of *L. reuteri DSM 17938* at a dose of 4 × 10^8^ CFU in hospitalized 69 Italian children aged six months to three years for acute diarrhoea, reduced significantly the frequency and the duration of diarrhoea on days 2 and 3, and also the number of children with diarrhoea on days 2 and 3, compared to the placebo without though affecting the duration of hospital stay [9]. In another multicenter, randomized, single-blinded, case control clinical trial [10] in 64 hospitalized children with acute watery diarrhoea, the administration of *L. reuteri DSM 17938* at a dose of 1 × 10^8^ CFU for five days was associated with reduction of the duration of diarrhoea and of the hospital stay as well as with better success rate on day 2 compared to 63 controls while, the same strain at a subsequent study in 60 children with acute diarrhoea presented at outpatients clinics, showed reduced duration of diarrhoea and better success rate on day two but no differences from the third day between the two groups [11]. Although the above strain has been solitary studied in childhood acute diarrhoea, the efficacy of the combined supplementation of ORS with *L. reuteri DSM 17938* and zinc in infants with acute diarrhoea, has not been studied so far.

The aim of the present study therefore, was to assess whether an ORS enriched with *L. reuteri DSM 17938* and zinc (ORS^+^Lr&Z) would be superior or equivalent to ORS without added probiotic and zinc (ORS^−^Lr&Z) in managing acute diarrhoea in well-nourished non-hospitalized infants and toddlers, and its effects on child’s and family’s normal activities

## 2. Patients and Methods

### 2.1. Study Design

The study was a randomized, double-blind, placebo-controlled study, conducted in patients with acute diarrhoea who were followed up at outpatient paediatric clinics in Athens, Greece during 30 months. The study protocol was approved by the ethics committee of the hospital and the study was registered at ClinicalTrials.gov (NCT 01886755), before the enrolment of the first patient. Informed consent was obtained from at least one parent or legal guardian prior to study inclusion.

### 2.2. Patients

Infants aged 6–36 months with acute diarrhoea defined as three or more watery or soft stools per day for the past 24–48 h and with mild to moderate degree of dehydration defined as one to four scores on Baily’s clinical dehydration scale [12], seen as outpatients were recruited. The exclusion criteria included the following: diarrhoea lasting more than 48 hours, clinical signs of severe dehydration defined as Bailey scale scores = or > 5, malnutrition defined as weight/height ratio below the 5th percentile, clinical signs of a coexisting severe acute systemic illness (meningitis, sepsis, pneumonia), immunodeficiency, severe chronic disease including cystic fibrosis, food allergy diagnosed by physician or other chronic gastrointestinal diseases, use of pre-/probiotics in the previous two weeks, use of antibiotics or any anti-diarrhoeal medication in the previous four weeks.

### 2.3. Methods

The patients were randomly assigned to receive either ORS^+^Lr&Z or ORS^−^Lr&Z of similar composition and osmolality (Table 1) both provided by BioGaia AB, Sweden. Oral rehydration took place over the first four hours while ongoing losses were replaced by administering 10 mL/kg of ORS using a graduated bottle provided by the sponsor, after each loose/watery stool or vomit until diarrhoea ceased or up to five days from the enrolment. Patients were allocated to each group according to a computer—generated randomisation using Random Allocation Software version 2.3.8 (StatsDirect Ltd., Chesire, UK). Treatment allocation was concealed to maintain the double-blind status.

The parents/legal guardians of the enrolled children were instructed to make daily records of the stools in a specific form using the Amsterdam stool scale (ISS) [13], the vomiting episodes, the volume of ORS consumed by the child on each day, other treatments or medications during the study period, adverse events, missed number of workdays for the parents and days at day care/nursery for the child, as well as any hospital admissions.

### 2.4. Outcome Measures

The primary outcome measures included the proportion of children without watery or soft (type A3–4 or B3–4 ISS) stools on day 2 of treatment, as well the time from the start of treatment up to the day of recording the last watery or soft stool. The secondary outcome measures included the reduction in the severity of diarrhoea assessed by the following: (a) the number of watery and soft (type A3–4 or B3–4 ISS) stools on each of the days during the study period; (b) the percentage of patients with watery or soft (type A3–4 or B3–4 ISS) stools on each day during the study period; and (c) the decrease in the diarrhoea severity score consisting of the sum of the points of three variables according to ISS: (i) number of bowel movements; (ii) type (A, B, C, and D) of stools and (iii) volume (1, 2, 3 and 4) of stools (Table 2); the number of vomiting episodes; the volume of ORS intake during the first day of treatment; the need for hospitalization; the loss of workdays for the parents; the loss of days from the day care/nursery for the children as well the need for medication administration due to diarrhoea.

### 2.5. Statistical Analysis

Continuous parameters are presented as mean and standard deviation (SD) and compared by non-parametric tests (Mann-Whitney) due to the small sample sizes and the extremely skewed distributions of outcome variables which are discrete and with relatively narrow range. Categorical variables are presented as absolute (n) and relative (%) frequencies and compared by the Fisher exact test. Mean differences (MD) of continuous outcomes between the two groups and 95% confidence intervals (CI) are reported, whereas absolute risk difference (ARD) with 95% CI were estimated for categorical outcomes. The level of statistical significance was set to 0.05. In cases of multiple comparisons, the Bonferroni correction was applied (0.05 divided by the number of comparisons). Data were analysed with Stata 11.2 MP statistical software (StataCorp, TX, USA).

## 3. Results

**Figure 1:**
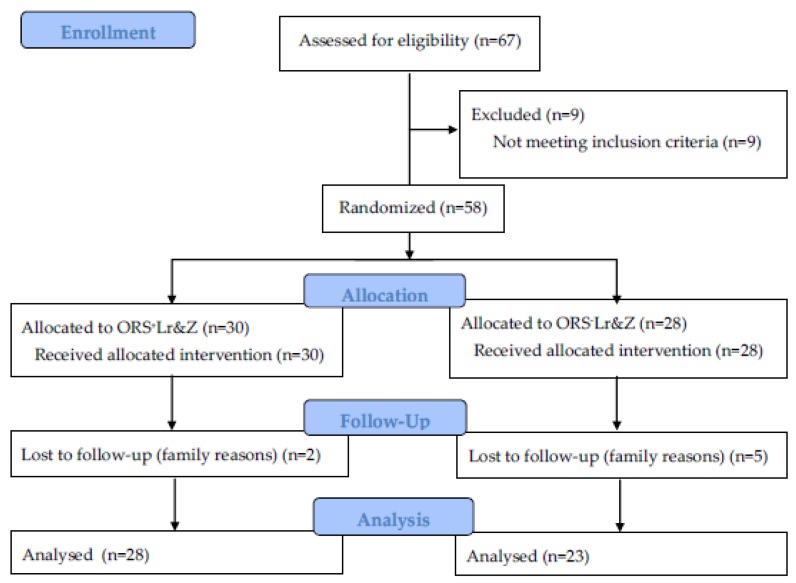
CONSORT flow diagram.

Fifty-eight children were randomly allocated to receive either ORS^+^Lr&Z (*n* = 30) or ORS^−^Lr&Z (*n* = 28) seven of whom (two from the ORS^+^Lr&Z and five from the ORS^-^Lr&Z group) were lost from follow up. A total of 51 children mean ± SD age 1.8 ± 0.7, 1.7 (1.3, 2.3), 37 males, 14 females, received either ORS^+^Lr&Z (*n* = 28, aged 1.7 ± 0.7, 21 males, seven females) or ORS^−^Lr&Z (*n* = 23, mean ± SD age 1.8 ± 0.7, 16 males, seven females) and were included in the analysis. The CONSORT flow diagram of the study is shown in Figure 1.

Age and gender were comparable between the two groups at baseline, as well as the severity of diarrhoea before recruitment (Table 3).

The proportion of children without diarrhoea on day two after the start of treatment did not differ significantly between the two groups: ORS^−^Lr&Z 13/23 (56.5%), ORS^+^Lr&Z 18/28 (64.3%), *p* = 0.8, ARD: 7.7% (−19.2%, 34.7%).

**Figure 2:**
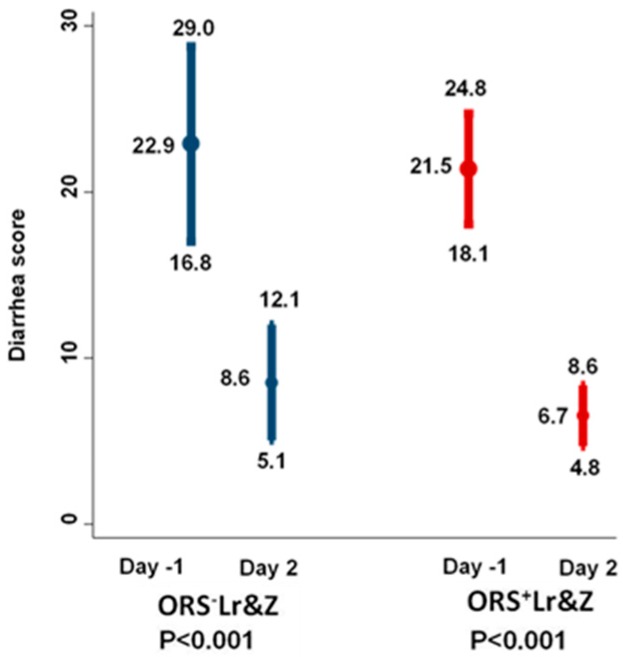
Improvement of the severity score of diarrhoea at day 2 compared to baseline (day^−1^). At each time point, the results are presented with means and 95% confidence intervals. Comparisons within groups were performed by the Wilcoxon signed-rank test for paired data, whereas data between groups were compared by the Mann-Whitney U test. Comparisons in the ORS^+^Lr&Z arm on day two versus day^−1^ (*p* < 0.001). Comparisons in the ORS^−^Lr&Z arm on day two versus day^−1^ (*p* < 0.001).

**Figure 3:**
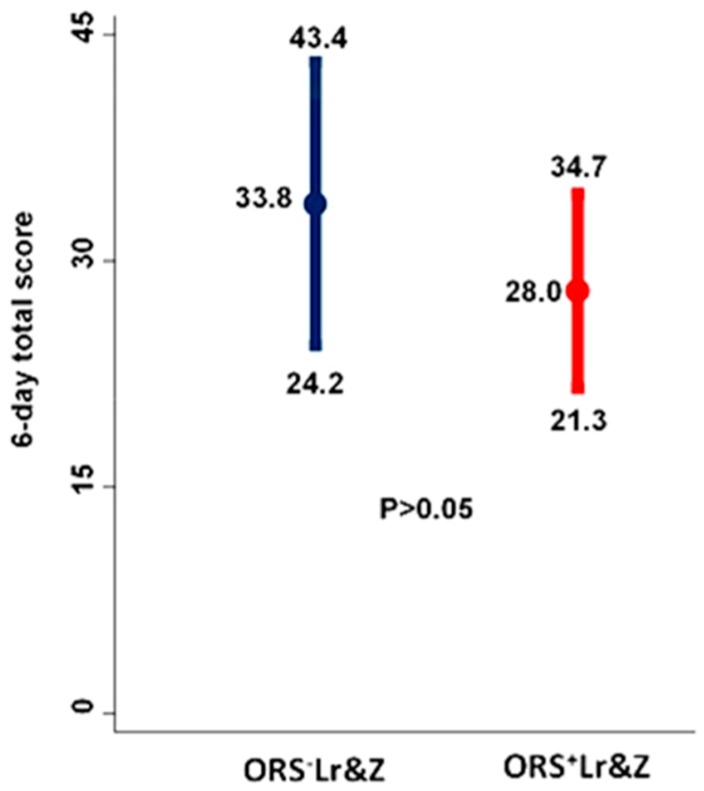
Total six-day severity score of diarrhoea. Results are presented with means and 95% confidence intervals. Comparisons between the ORS^+^Lr&Z and the ORS^-^Lr&Z arms were performed by the Mann-Whitney U test (*p* > 0.5).

All of the outcomes in the intention to treat analysis showed a trend to be better in the ORS^+^Lr&Z group, however statistical significance was not reached in any of them. Both groups showed comparable improvement in the severity of diarrhoea on day two following the start of treatment (Table 4, Figure 2) as well as during the study period (Table 4, Figure 3).

Both ORS with or without supplementation with *L. reuteri DSM 17938* and zinc managed to decrease significantly the severity of diarrhoea on day 2, based on the severity score that took into account both stool consistency and volume according to ISS (Figure 2).

Furthermore, although the ORS supplemented with *L. reuteri DSM 17938* and zinc had a tendency to achieve a greater decrease in the 6-day severity score of diarrhoea, the difference between the two groups did not reach statistical significance (Figure 3).

**Figure 4:**
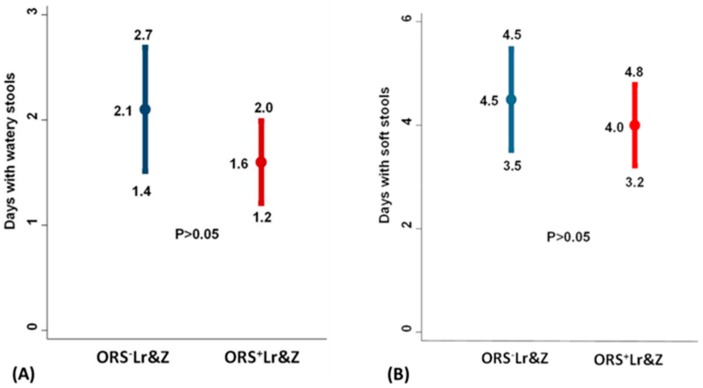
(**A**): Number of days with watery stools. (**B**): Number of days with soft stools. Results are presented with means and 95% confidence intervals. Comparisons between the ORS^+^Lr&Z and the ORS^-^Lr&Z arms were performed by the Mann-Whitney U test (*p* > 0.5).

Similarly, although a tendency was seen in the group receiving ORS supplemented with *L. reuteri DSM 17938* and zinc to have smaller duration of watery diarrhoea as well as of soft stools compared to the group which received non-supplemented ORS, again, the difference did not reach statistical significance (Figure 4A,B).

**Figure 5:**
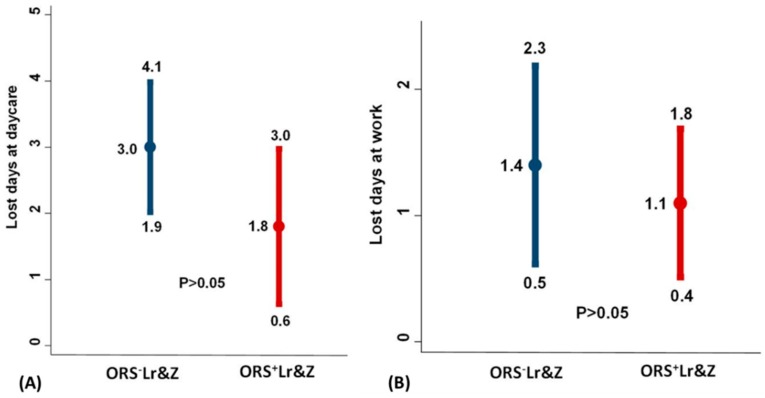
(**A**): Number of lost day care/nursery days for patients. (**B**): Number of lost work days for parents. Results are presented with means and 95% confidence intervals. Comparisons between the ORS^+^Lr&Z and the ORS^-^Lr&Z arms were performed by the Mann-Whitney U test (*p* > 0.5).

The same was true for the number of days lost from the day care for the infants and from work for the parents, which tended to be less in the group which received ORS supplemented with *L. reuteri DSM 17938* and zinc, but again, the differences did not reach statistical significance (Figure 5A,B).

The number of vomiting episodes was comparable between the ORS^−^Lr&Z and the ORS^+^Lr&Z groups at baseline: mean ± SD, median (IQR) 0.09 ± 0.28, 0 (0, 0) vs. 0.07 ± 0.26, 0 (0, 0) respectively, *p* = 0.4, while no vomiting episodes were recorded during the study period in any of the two groups.

The consumed volume of ORS during the first 24 h did not differ between the ORS^+^Lr&Z and the ORS^−^Lr&Z groups: mean ± SD, median (IQR) 309.8 ± 235.3, 265 (120, 415) vs. 326.5 ± 195.9 respectively, 300 (170, 490), *p* = 0.5.

None of the patients was hospitalized during the study period and no other medications for diarrhoea or antibiotics were administered by any of the patients.

## 4. Discussion

This study is the first study assessing the efficacy of an ORS supplemented with *L. reuteri DSM 17938* and zinc in infants with acute diarrhoea. Furthermore, this is the first study that uses for the assessment of the efficacy of the above ORS supplementation, an objective measure (ISS), that takes into account both consistency and volume of the infant’s stools. In addition, this is the first study assessing the effects of the above combination, on child’s and family’s normal activities during acute diarrhoea. We showed that ORS^+^Lr&Z and ORS^−^Lr&Z were both associated with reduction in the severity of diarrhoea two days following the start of treatment in a group of well-nourished, non-hospitalized infants and toddlers with acute gastroenteritis. Furthermore, we showed that all of the outcomes including the severity of diarrhoea based on ISS, the duration of watery diarrhoea, the percentages of infants with no diarrhoea on days 3 and 5 as well as the number of absences from the day care for the infants and the parents from the work, all showed a trend to be better in the ORS^+^Lr&Z group without though reaching statistical significance, probably due to the relatively small number of recruited patients.

Other studies using probiotic strains such as *L. reuteri ATCC 55730*, *Lactobacillus rhamnosus 19070-2* and *L. reuteri DSM 12246*, reported beneficial effects in children with acute gastroenteritis [3,4,5,6,7,14]. *Lactobacillus rhamnosus 19070-2* and *L. reuteri DSM 12246* were both effective in reducing the duration of diarrhoea after intervention in hospitalized and non-hospitalized children with mild diarrhoea while, a greater efficacy was noted in case of early (<60 hours after the start of diarrhoea) intervention [4,5]. Furthermore, *L. reuteri DSM 17938* and *L. reuteri ATCC 55730* were reported in one meta-analysis in hospitalized children 3 to 60 months (*n* = 196 and *n* = 156 respectively), to reduce significantly the duration of diarrhoea compared to placebo or no treatment respectively [15].

The mechanisms of the effect of *L. reuteri* in acute diarrhoea are not fully understood. It has been shown that *L. reuteri* can produce reuterin, a broad-spectrum antimicrobial agent [16], which may be responsible for the inhibition of pathogenic microorganisms in the gastrointestinal tract, but also, it can decrease intestinal permeability [17,18] and stimulate the intestinal immune responses [19,20]. Furthermore, *L. reuteri DSM 17938* and *ATCC PTA 6475* were shown in a model of rotavirus infection in new-born mice, to increase the early mucosal rotavirus-specific IgA as well as the diversity of the distal gut microbiome, attenuating rotavirus induced enteritis and reducing the duration of diarrhoea by one day. In our study, we did not perform gut microbiome analysis in the recruited infants with acute diarrhoea. Therefore, we cannot draw any conclusions from this study on the possible effects of the supplemented ORS with *L. reuteri DSM 17938* and zinc on infants distal gut microbiome during acute diarrhoea. Other preliminary studies suggested that *L. reuteri 17938* increased the number of villus goblet cells bearing markers of intestinal stem cell activity [21,22,23].

Based on the available evidence, the European Society for Pediatric Gastroenterology, Hepatology and Nutrition recommended the use of *Lactobacillus rhamnosus GG*, *Saccharomyces boulardii*, *L. reuteri DSM 17938*, and the heat-inactivated *Lactobacillus acidophilus LB* as a supplement of the ORS for the management of childhood acute gastroenteritis [24].

With regards to zinc supplementation of ORS in children with acute diarrhoea, it has been shown to be beneficial in malnourished children [25] but has been poorly studied in well-nourished ones. The postulated mechanisms include improved absorption of water and electrolytes by the intestine [26,27,28], regeneration of gut epithelium [29], increased levels of enterocyte brush-border enzymes [30], and enhanced immunologic mechanisms. Therefore, WHO recommended early oral rehydration therapy and zinc supplementation to treat acute diarrhoea in children [1]. Our study failed to show superiority of the enriched with zinc ORS in the study group consisting of well-nourished infants with mild diarrhoea not requiring hospitalization, probably due to the fact that the study was underpowered due to the relatively small number of recruited patients.

The main limitation of the study was, therefore, the relatively small number of the recruited children. The fact, however, that the study was a well-designed, double blind, placebo-controlled trial, the first that assessed an ORS enriched with *L. reuteri DSM 17938* and zinc in infants with acute gastroenteritis, and also used for the assessment of the severity of diarrhoea an objective method (ISS), make the results a valuable contribution to the meta-analyses on the topic, which has clinical importance for health professionals, including general practitioners, general pediatricians, pediatric gastroenterologists, and nutritionists.

## 5. Conclusions

This randomised, double blind, placebo-controlled trial showed that ORS supplemented with *L. reuteri DSM 17938* and zinc had comparable efficacy with ORS of similar composition and osmolality without added probiotic and zinc, in managing acute diarrhoea, in well-nourished, non-hospitalised infants and toddlers with acute diarrhoea. ORS enriched with *L. reuteri DSM 17938* and zinc was well tolerated without adverse effects.

## Figures and Tables

**Table 1:** Composition of the study products.

	ORS Enriched with *L. reuteri DSM 17938* and Zinc (1 Sachet)	ORS without *L. reuteri DSM 17938* and Zinc (1 Sachet)
Protein	<0.1 g	<0.1 g
Carbohydrates	3.75 g	3.75 g
of which glucose	3.75 g	3.75 g
Fat	<0.1 g	<0.1 g
Sodium	0.35 g/15 mmol	0.35 g/15 mmol
Chloride	0.4 g/11 mmol	0.4 g/11 mmol
Potassium	0.2 g/5 mmol	0.2 g/5 mmol
Citrate	0.5 g/3 mmol	0.5 g/3 mmol
Zinc	1.5 mg/0.02 mmol	0 mg/0 mmol
Osmolality	220 mOsm/kg H_2_O	220 mOsm/kg H_2_O
*L. reuteri DSM 17938*	1 × 10^9^ CFU (Colony Forming Units)	0

**Table 2:** Total score of severity of diarrhoea based on Amsterdam stool scale.

Variable									
		Consistency				Volume			
Category		A	B	C	D	1	2	3	4
Points	1 point for each bowel movement	2	1	0	0	1	2	3	4

The total score consists of the sum of the product of the points of three variables: (i) number of bowel movements; (ii) Type (A, B, C, and D) of stools and iii) Volume (1, 2, 3, and 4) of stools.

**Table 3:** Baseline characteristics in the two study arms.

	ORS^−^Lr&Z	ORS^+^Lr&Z	*p*-Value
Age (years) *	1.8 ± 0.7, 1.8 (1.3, 2.4)	1.7 ± 0.7, 1.6 (1.3, 2.1)	0.5 **
Severity of diarrhoea (score) *	4.7 ± 3.6, 5 (2, 6)	4.3 ± 3.1, 3.5 (2.0, 6.5)	0.6 **
Gender, males/females, *n* (%)	16/7 (69.6%/30.4%)	21/7 (75.0%/25%)	0.8 ***

* Mean ± SD, median (IQR); ** Mean (95% confidence interval); *** Mann-Whitney test, level of significance after Bonferroni correction: 0.01.

**Table 4:** Resolution of diarrhoea during the study period.

	Number of Watery or Soft (type A3–4 or B3–4 ISS ^#^) Stools *				Proportion of Patients without Diarrhoea			
	ORS^−^Lr&Z	ORS^+^Lr&Z	MD (ORS^+^Lr&Z vs. ORS^−^Lr&Z **	*p*-Value ***	ORS^−^Lr&Z	ORS^+^Lr&Z	ARD ** (ORS^+^Lr&Z vs. ORS^−^Lr&Z)	*p*-Value ****
Day 1	1.17 ± 1.85 0 (0, 2)	0.96 ± 1.13 1 (0, 2)	−0.21 (−1.05, 0.63)	0.9	12/23 (52.2%)	13/28 (46.4%)	−5.7% (−33.2%, 21.8%)	0.8
Day 2	0.74 ± 1.00 0 (0, 1)	0.64 ± 1.00 0 (0, 1)	−0.10 (−0.66, 0.46)	0.6	13/23 (56.5%)	18/28 (64.3%)	7.7% (−19.2%, 34.7%)	0.8
Day 3	0.69 ± 0.87 0 (0, 2)	0.46 ± 0.83 0 (0, 1)	−0.23 (−0.71, 0.25)	0.3	13/23 (56.5%)	20/28 (71.4%)	14.9% (−11.1%, 41.2%)	0.4
Day 4	0.34 ± 0.71 0 (0, 1)	0.39 ± 0.62 0 (0, 1)	0.05 (−0.33, 0.42)	0.6	17/23 (73.9%)	19/28 (67.9%)	6.1% (−31.0%, 18.9%)	0.8
Day 5	0.30 ± 0.47 0 (0, 1)	0.14 ± 0.52 0 (0, 0)	−0.16 (−0.44, 0.12)	0.05	16/23 (69.6%)	26/28 (92.9%)	23.3% (2.0%, 44.5%)	0.06

^#^ Infant Stool Scale; * Mean ± SD, median (IQR); ** Mean (95% confidence interval); *** Mann-Whitney test, level of significance after Bonferroni correction: 0.01; **** Fisher’s exact test, level of significance after Bonferroni correction: 0.01.

## Abbreviations

ORS	Oral rehydration solution
*L. reuteri*	Lactobacillus reuteri
ORS^+^Lr&Z	ORS enriched with *L. reuteri DSM 17938* and zinc
ORS^−^Lr&Z	ORS of similar composition and osmolality without added probiotic and zinc
MD	Mean differences
SD	Standard deviation
95% CI	95% confidence intervals
